# Factors Affecting Student Anxiety estimated by linear regression

**DOI:** 10.1192/j.eurpsy.2024.868

**Published:** 2024-08-27

**Authors:** T. Urtnasan, N. Namdag

**Affiliations:** ^1^Mental health, Etugen, Ulaanbaatar, Mongolia

## Abstract

**Introduction:**

This study was conducted to estimate anxiety levels at the university level to address the issues of changing the healthy lifestyle of students, promoting health, spending their free time properly, being healthy, and developing good habits.

**Objectives:**

The aim is to study the factors affecting student anxiety.

**Methods:**

The study was conducted by random sampling of 1356 students from the 1st to 5th year of the medical school of Etugen University in the academic years 2020-2022.

**Results:**

4.3%(58) of students’ inherent anxiety was low, 62.3%(845) was moderate, and 33.4%(453) were high, while 4.2%(57) had low anxiety and 42.4% (575) were anxious due to the student’s situation. had moderate anxiety and 53.4% (724) had high anxiety.

According to the research, 69.9% (948) had a low level of motivation, 23.4% (317) had a below-average level, 5.6% (76) had an average level, and 1.1% (15) had an above or higher level.

There is a weak inverse relationship between congenital anxiety and the course of study (r=-0.054*), and a weak inverse relationship between age (r=-0.048). There is a weak (r=-0.125**) inverse relationship between situational anxiety and the course of study, and a weak (r=-0.127**) inverse relationship between age.

When examining the relationship between students’ natural anxiety and the factors influencing it, there is a moderate (r=0.630**) direct correlation between natural anxiety and situational anxiety.

According to the one-factor linear regression analysis of students’ congenital anxiety (B=-1.964; 95%CI (-3.07 - 0.858); p<0.01), increasing the age by one increases congenital anxiety by 1.964 times, p<0.01, which means that the linear model is good. indicating a match.

Multivariate linear regression analysis showed that situational anxiety (B=3.845; 95%CI (6.288 - 347.90); p<0.01) or a one-level increase in situational anxiety increased congenital anxiety 3.845 times p<0.00. is relevant

**Conclusions:**

According to the multivariate analysis, there is a linear significant relationship between one increase in student age, and -1.256-fold decrease in situational anxiety, and a -5.464-fold decrease in situational anxiety when not suffering from mental illness.

**Disclosure of Interest:**

None Declared

